# The potential relevance of the endocannabinoid, 2-arachidonoylglycerol, in diffuse large B-cell lymphoma

**DOI:** 10.18632/oncoscience.289

**Published:** 2016-01-30

**Authors:** Jianqing Zhang, Daniel Medina-Cleghorn, Leon Bernal-Mizrachi, Paige M. Bracci, Alan Hubbard, Lucia Conde, Jacques Riby, Daniel K. Nomura, Christine F. Skibola

**Affiliations:** ^1^ Department of Epidemiology, School of Public Health, Comprehensive Cancer Center, University of Alabama at Birmingham, AL, USA; ^2^ Department of Nutritional Sciences and Toxicology, University of California at Berkeley, CA, USA; ^3^ Winship Cancer Institute of Emory University, Atlanta, GA, USA; ^4^ Department of Epidemiology & Biostatistics, School of Medicine, University of California at San Francisco, CA, USA; ^5^ School of Public Health, Division of Biostatistics, University of California at Berkeley, CA, USA

**Keywords:** endocannabinoids, 2-AG, DLBCL, lymphoma, obesity

## Abstract

Diffuse large B-cell lymphoma is an aggressive, genetically heterogenerous disease and the most common type of non-Hodgkin lymphoma among adults. To gain further insights into the etiology of DLBCL and to discover potential disease-related factors, we performed a serum lipid analysis on a subset of individuals from a population-based NHL case-control study.

An untargeted mass-spectrometry-based metabolomics platform was used to analyze serum samples from 100 DLBCL patients and 100 healthy matched controls. Significantly elevated levels of the endocannabinoid, 2-arachidonoylglycerol (2-AG), were detected in the serum of DLBCL patients (121%, *P <* 0.05). In the male controls, elevated 2-AG levels were observed in those who were overweight (BMI ≥ 25 - < 30 kg/m2; 108%, *P* < 0.01) and obese (BMI ≥ 30 kg/m^2^; 118%, *P* < 0.001) compared to those with a BMI < 25 kg/m^2^. DLBCL cell lines treated with exogenous 2-AG across a range of concentrations, exhibited heterogenous responses: proliferation rates were markedly higher in 4 cell lines by 22%-68% (*P* < 0.001) and lower in 8 by 20%-75% (*P* < 0.001). The combined findings of elevated 2-AG levels in DLBCL patients and the proliferative effects of 2-AG on a subset of DLBCL cell lines suggests that 2-AG may play a potential role in the pathogenesis or progression of a subset of DLBCLs.

## INTRODUCTION

Diffuse large B-cell lymphoma (DLBCL) is a cancer of B-lymphocytes and is the most common type of non-Hodgkin lymphoma (NHL) among adults [1]. Approximately 30–40% of DLBCL patients will present with localized stage I or II disease at the time of diagnosis, whereas the remainder will have more widespread advanced disease [2]. Established risk factors include severe immunosuppression and rare genetic conditions [3], and there is mounting evidence to support the important roles of obesity, diet, history of autoimmune disease and occupational exposures [4–6]. To gain further insights into the etiology of DLBCL and to discover potential disease-related factors in blood, we performed an untargeted and unbiased lipid metabolite analysis of serum from 100 DLBCL patients collected prior to any chemotherapy, and 100 healthy controls. We found statistically significant higher levels of the endogenous cannabinoid (endocannabinoid) signaling lipid, 2-arachidonoylglycerol (2-AG), in the serum of DLBCL patients compared to matched controls. *In vitro* studies were conducted in 16 DLBCL cell lines to further explore the relevance of 2-AG in DLBCL.

## RESULTS

### Serum lipidomic profiling of DLBCL cases and controls

**Figure 1:**
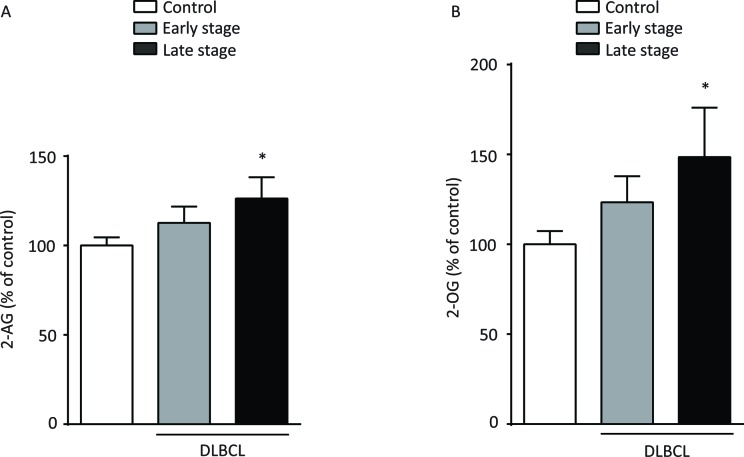
**A.** Serum 2-AG and **B.** 2-OG levels in early stage (*n* = 42) and late (*n* = 58) stage DLBCL cases compared to controls (*n* = 100). Data are expressed as the percent of controls, mean ± SE. *P* < 0.05.

Lipidomic profiling revealed significant elevated levels of 2-AG in the serum of DLBCL cases (121 ± 8%, *P <* 0.05) compared to controls. The results did not vary by sex. When cases were stratified by stage, serum 2-AG levels were significantly higher in DLBCL cases with late stage disease (126 ± 12%, *P* < 0.05) compared to controls (Figure 1A). We also found significantly increased levels of the endocannabinoid-like lipid, monoacylglycerol 2-oleoylglycerol (2-OG), in the serum of DLBCL cases (138 ± 17%, *P* < 0.05) compared to controls that did not vary by sex. When cases were stratified by stage, serum 2-OG levels were significantly higher in DLBCL cases with late stage disease (149 ± 27%, *P* < 0.05) compared to controls (Figure 1B).

### Elevated levels of 2-AG in controls with high body mass index (BMI)

**Figure 2:**
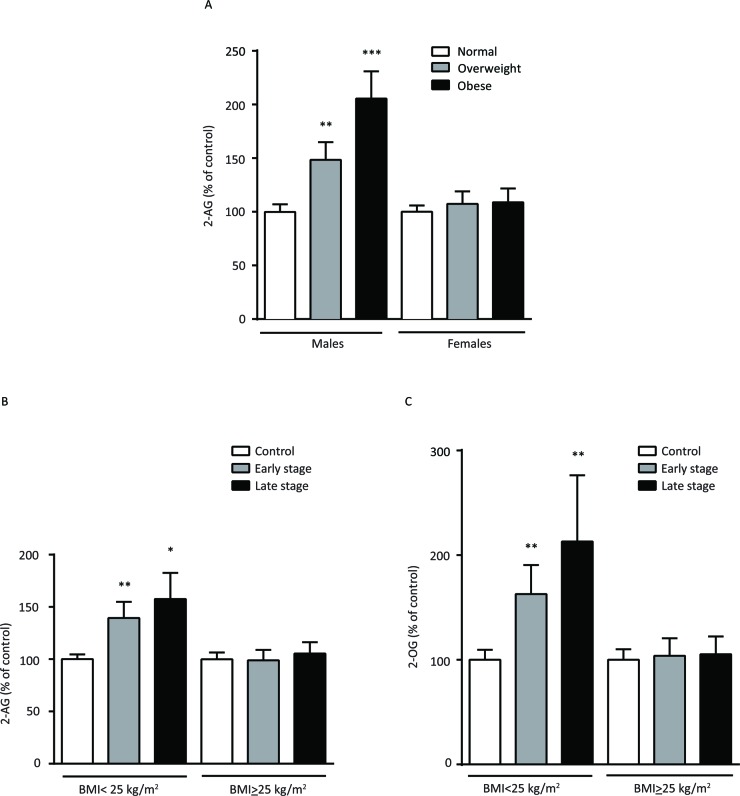
**A.** Serum 2-AG levels in healthy overweight (BMI ≥ 25 - < 30 kg/m2; *n* = 18 males, *n* = 13 females) and obese (BMI ≥ 30 kg/m^2^; *n* = 11 males, *n* = 10 females) controls as a percent of those with a BMI < 25 kg/m^2^; *n* = 21 males, *n* = 27 females); and **B.** serum 2-AG and **C.** 2-OG levels of DLBCL patients by stage (early stage, *n* = 20 males, *n* = 22 females; later stage, *n* = 28 males, *n* = 30 females) as a percent of healthy control subjects by BMI < 25 kg/m2 and ≥ 25 kg/m^2^. Data are expressed as mean ± SE. * *P* < 0.05, ** *P* < 0.01, *** *P* < 0.001.

Previous studies reported elevated 2-AG levels in obese mice and humans [7–10]; thus, we assessed 2-AG levels by BMI in the controls. Here we found that 2-AG levels were positively correlated with BMI only in men, where overweight (BMI ≥ 25 - < 30 kg/m^2^; 148 ± 17%, *P* < 0.01) and obese subjects (BMI ≥ 30 kg/m^2^; 206 ± 25%, *P* < 0.001) had higher 2-AG levels compared to those with BMI < 25 kg/m^2^ (Figure 2A). We did not find any correlation with 2-OG levels and BMI in men or women.

### Elevated levels of 2-AG in the serum of DLBCL cases with BMI < 25 kg/m^2^

**Figure 3:**
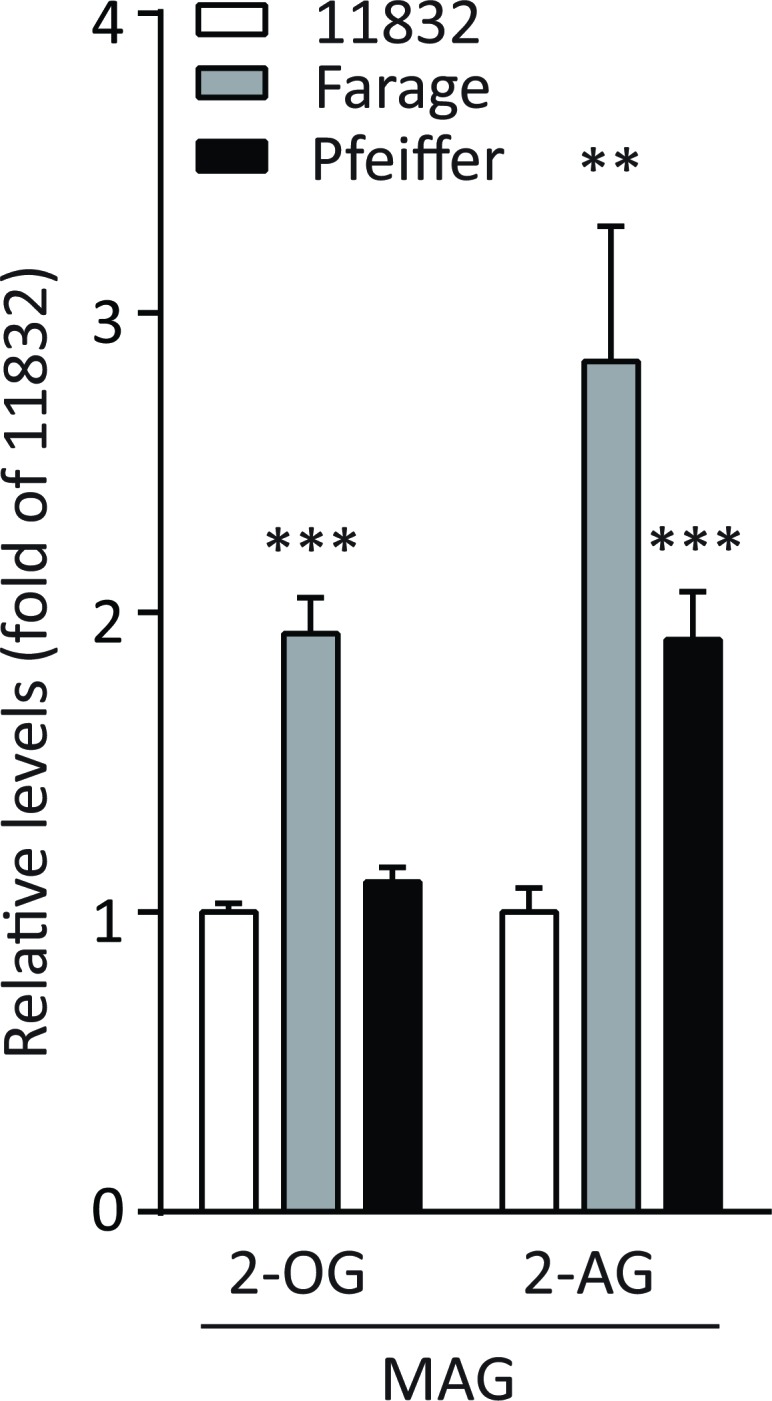
Monoacylglycerol (MAG) levels in cell lysates of untreated Pfeiffer, Farage and LBCL11832 cells Data are from experiments performed in quadruplicate and expressed as fold changes compared to LBCL11832. Data are expressed as mean percentage of the controls, ± SE. ** *P* < 0.01, *** *P* < 0.001 vs. LBCL11832.

In DLBCL cases, we found no significant differences in 2-AG levels when the analysis was stratified by BMI groups. However, in an analysis restricted to those with BMI < 25 kg/m^2^, we found significantly higher 2-AG levels in early stage (140 ± 19%, *P* < 0.01) and later stage DLBCL cases (158 ± 25%, *P* < 0.01) compared to healthy controls (Figure 2B). We also found significantly higher levels of 2-OG in early stage (163 ± 28%, *P* < 0.01) and later stage DLBCL cases (213 ± 63%, *P* < 0.05) compared to controls (Figure 2C). We observed relatively consistent findings in a lipidomic analysis of untreated DLBCL Farage and Pfeiffer cell lines. When compared to the lymphoblastoid cell line, LBCL11832, we found a 2.8- and 1.9-fold increase in 2-AG levels in Farage (*P* < 0.001) and Pfeiffer (*P* < 0.001) cells, respectively, and a 1.9- fold increase in 2-OG levels in Farage cells (*P* < 0.001) (Figure 3). Significant changes in additional lipids in Farage and Pfeiffer cell lines are presented in Figure S1.

### Effects of 2-AG on DLBCL cell proliferation

**Figure 4:**
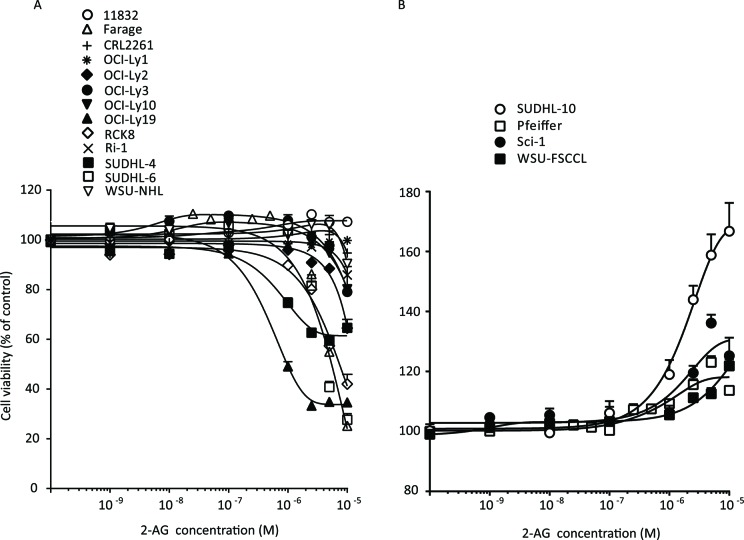
Concentration-dependent effects of 2-AG on cell viability in A Farage, OCI-Ly2, OCI-Ly3, OCI-Ly10, OCI-Ly19, RAC8, SUDHL-4, SUDHL-6, and LBCL11834 and **B.** SUDHL-10, Sci-1, Pfeiffer and WSU-FSCCL. All cells were treated with 2-AG at concentrations 1 nM to 10 μM.

We next investigated the effects of 2-AG on cell proliferation in 16 DLBCL cell lines at concentrations ranging from 1 nM to 10 μM. At physiologically relevant concentrations (≤1 μM) [11, 12], 2-AG stimulated increased proliferation of Farage by 7.9% (*P* < 0.001); Pfeiffer, 10% (*P* < 0.001); OCI-Ly-3, 8.1% (*P* < 0.001); OCI-Ly-10, 4.3% (*P* < 0.01); Sci-1, 6.3%, (*P* < 0.01); SUDHL-6, 4.0% (*P* < 0.01); SUDHL-10, 19% (*P* < 0.001); and WSU-FSCCL, 5.8% (*P* < 0.001) compared to the vehicle-treated controls (Figure 4A, 4B). Similar effects of 2-AG were found on cell proliferation of LBCL11832 cells (7.1%, *P* < 0.01) (Figure 4A).

Higher concentrations of 2-AG (>1 μM) led to more marked increases in the proliferation of SUDHL-10, Sci-1, Pfeiffer and WSU-FSCCL cells in a concentration-dependent manner (Figure 4B). For example, at 5 μM, 2-AG increased proliferation of SUDHL10 by 67%, (*P* < 0.001), Sci-1 by 36% (*P* < 0.001), Pfeiffer by 23% (*P* < 0.001) and WSU-FSCCL by 22% (*P* < 0.001). No apparent changes were observed on cell proliferation of LBCL11832 cells with increased concentration of 2-AG (Figure 4A). However, we observed moderate antiproliferative effects of 2-AG at 10 μM in WSU-NHL, 5.8% (*P* < 0.001); OCI-Ly1, 0.3% (*P* > 0.1); Ri-1, 14.2% (*P* < 0.001) and CRL-2261, 5.3% (*P* < 0.001) cell lines (Figure 4A), and strong antiproliferative effects in OCI-Ly3, 21% (*P* < 0.001); OCI-Ly10, 19.8% (*P* < 0.001); OCI-Ly2, 36.5% (*P* < 0.001); RCK8, 58.9% (*P* < 0.001); Farage, 74.8% (*P* < 0.001); SUDHL-6, 72.3% (*P* < 0.001); SUDHL-4, 35.4% (*P* < 0.001); and OCI-Ly19, 65.3% (*P* < 0.001) cells (Figure 4A).

The effects of 2-AG on cell proliferation were also confirmed by direct counting of the viable cells (trypan blue exclusion) and BrdU incorporation (data not shown). We did not find that the anti- or pro-proliferative effects of 2-AG were correlated with whether the DLBCL subtype was either germinal center or activated B cell-like.

### Effects of enzyme inhibitors involved in the breakdown of 2-AG on induced proliferation of DLBCL cell lines

To investigate the possible mechanism of 2-AG-induced cell proliferation, we first excluded the possiblility of the involvement of the cannabinoid receptors, CB1 and CB2, by treating SUDHL-10, Sci-1, Pfeiffer, and WSU-FSCCL cells with CB1 and CB2 agonists (CP47497, AM1241, Win-55–212-2, and CP55940) or pretreated cells with antagonists (Rimonabant, AM251, SR144528). No proliferative effects or attenuation of the proliferative effects was found (data not shown).

**Figure 5:**
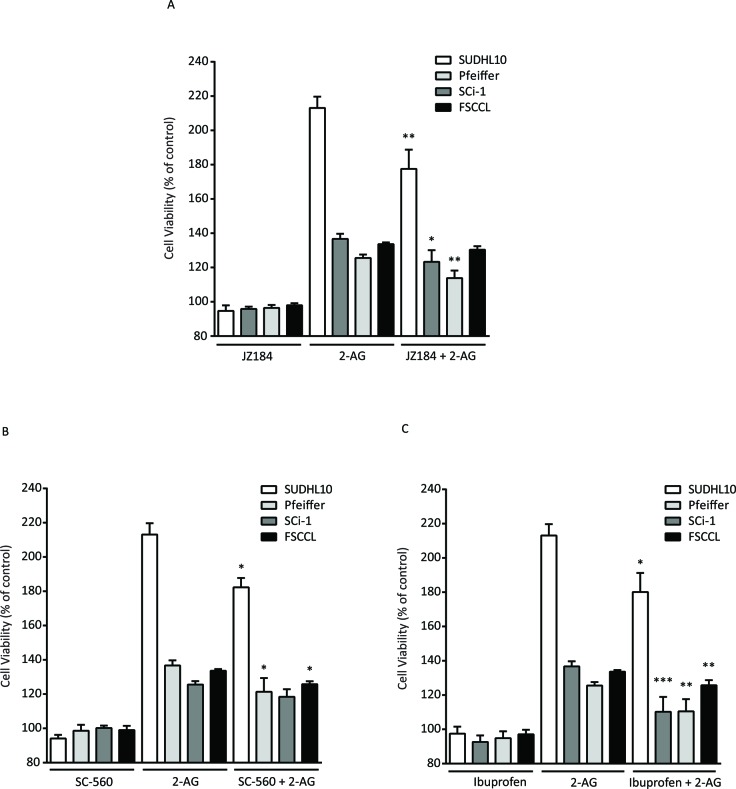
Effects of A. the MAGL inhibitor, JZ184 (1 μM), and the COX inhibitors, B. SC560 (1 μM) and C. Ibuprofen (40 μM), on 2-AG-induced proliferation of SUDHL-10, Sci-1, Pfeiffer and WSU-FSCCL cells Data are expressed as mean ± SE. * *P* < 0.05, ** *P* < 0.01, *** *P* < 0.001 compared with 2-AG treatment without inhibitor.

2-AG can be hydrolyzed by MAGL to arachidonic acid which, in turn, is metabolized to eicosanoid signaling lipids through cyclooxygenases; therefore, we treated SUDHL-10, Sci-1, Pfeiffer, and WSU-FSCCL cells with MAGL and COX inhibitors to determine whether we could reverse the pro-proliferative effects of 2-AG. Pre-treatment of cell lines with the MAGL inhibitor, JZ184 (1 μM), showed modest statistically significant decreased cell growth in Pfeiffer (11%, *P* < 0.01), Sci-1 (13%, *P* < 0.05), and SUDHL-10 (36%, *P* < 0.01) cells (Figure 5A). Pre-treatment of cell lines with the COX1 selective inhibitor, SC-560 (1 μM), resulted in decreased cell growth in Sci-1 (15%, *P* < 0.05), FSCCL (7.8%, *P* < 0.001) and SUDHL-10 (31%, *P* < 0.01; Figure 5B). Pre-treatment of cell lines with the non-selective, reversible COX inhibitor, Ibuprofen (40 μM), showed significant decreased cell growth in Pfeiffer (15%, *P* < 0.01), Sci-1 (27%, *P* < 0.001), FSCCL (7.9%, *P* < 0.01), and SUDHL-10 (33%, *P* < 0.01) compared to 2-AG-treated cells (Figure 5C).

### Expression of endocannabinoid system-related genes in DLBCL cell lines

Cannabinoid receptor-1 (*CNR1*), *CNR2*, G-protein-coupled receptor 55 (*GPR55*), fatty acid amide hydrolase (*FAAH*), monoacylglycerol lipase (*MAGL*), cyclooxygenase -1 (*COX1*) and *COX2* gene expression levels were ascertained to determine if expression levels correlated with the proliferative response of DLBCL cell lines to 2-AG. We found that the Pfeiffer, SUDHL-10 and WSU-FSCCL cell lines that exhibited a high proliferative response to 2-AG at 5 μM showed 2- to 3-fold lower *CNR1* expression in comparison to the the cell lines, Farage, SUDHL-4, SUDHL-6, WSU-NHL, Ri-1 and OCI-Ly19 that exhibited a minimal or anti-proliferative response to 2-AG at the same concentration (Figure S2). No other patterns were observed related to *CNR2*, *GPR55*, *FAAH*, *MAGL*, *COX1* and *COX2* gene expression in the DLBCL cell lines and their response to 2-AG.

### Effects of charcoal/dextran treated FBS on 2-AG stimulated proliferation of DLBCL cell lines

To minimize the effects of the endocannabinoids potentially present in normal FBS, we cultivated the cells in RPMI media supplemented with charcoal/dextran-treated FBS (CFBS) for 24 hours before and after treatment. The cell line most responsive to 2-AG, SUDHL-10, was used to investigate the effects of normal FBS versus CFBS and of different CFBS concentrations on the actions of 2-AG on cell proliferation. We observed stronger proliferative effects of 2-AG (10%, *P* < 0.001) on cells treated in RPMI media supplemented with CFBS versus FBS media (Figure S3A). Furthermore, a minimum of 1% of CFBS was necessary to support the survival of cell lines and 5% of CFBS was necessary to have maximum 2-AG-induced cell proliferation in 4 days (Figure S3B).

## DISCUSSION

In an untargeted lipidomic analysis, we found significantly higher 2-AG levels in the serum of DLBCL cases compared to healthy controls. Moreover, the levels of 2-AG also were positively correlated with stage of the disease, which is consistent with reports that high 2-AG plasma concentrations are associated with tumor progression in mice and in some human cancers [13]. In a stratified analysis, 2-AG levels were higher in DLBCL cases with a BMI < 25 kg/m^2^ compared to matched controls, whereas no differences in 2-AG levels were found when the analysis was restricted to the overweight/obese groups. The pre-diagnostic weight loss inherently seen in many DLBCL patients may have precluded us from detecting a positive association between 2-AG levels and BMI. Levels of 2-OG, a monoacylglycerol that shares the same biosynthetic and catabolic pathways as 2-AG, also were elevated in the serum of DLBCL cases and were positively correlated with stage of the disease. However, there was no correlation between 2-OG levels and BMI in men or women. A lipidomic analysis of Farage and Pfeiffer cell lines revealed that 2-AG levels were approximately two-fold higher than in lymphoblastoid cells, and 2-OG two-fold higher in Farage cells providing further support for a role of abnormal lipid metabolism in cancer development and progression [14] that may be relevant to DLBCL. Further studies are needed to confirm whether primary DLBCL cells from patients also exhibit elevated 2-AG and 2-OG levels.

Links between obesity and risk of multiple types of cancers [15, 16] including DLBCL [6, 17, 18] have been well established. Moreover, there is mounting evidence that the endocannabinoid system has emerged as a major signaling network to control central aspects of food intake and energy homeostasis [19]. Endocannabinoids and the cannabinoid receptors are deregulated in animal and human obesity, and might occur in a gender- and tissue-specific manner [19, 20]. Fanelli *et al*. reported that circulating 2-AG levels were ∼20% higher in men than in women [21]. Our finding of higher 2-AG levels in sera from overweight/obese men, but not women, supports previous reports of a positive correlation between 2-AG levels and body weight in men [22]. Our results raise the question of whether higher serum 2-AG levels in obese men may be mechanistically relevant in the pathogenesis of DLBCL.

Cannabinoids and modulators of the endocannabinoid system produce anti-proliferative, anti-metastatic, anti-angiogenic, and pro-apoptotic actions in a broad spectrum of tumors [23–26]. However, cannabinoids may induce cell growth depending on cancer cell type, the specific cannabinoid/endocannabinoid compounds, and metabolite concentrations. DeMorrow *et al*. demonstrated that 2-AG stimulated cholangiocarcinoma cell proliferation at 10 μM [27, 28]. Low doses of two synthetic cannabinoids and tetrahydrocannabinol enhanced B-cell growth at nanomolar concentrations; the proliferative effect was exerted via the CB2 receptor, CNR2, in the peripheral blood and in tonsilar B-cells [29, 30]. It was suggested that increased levels of circulating 2-AG in serum may favor tumor progression in a mouse melanoma model [13]. It is interesting to note that we also observed that 2-AG exerted disparate effects *in vitro* in a concentration- and DLBCL cell-line-specific manner. Specifically, we found that *CNR1* expression was 2- to 3-fold lower in the group of DLBCL cell lines that exhibited increased proliferation in response to 2-AG (Figure S2) compared to the majority of cell lines that showed an antiproliferative response. Our study suggests that 2-AG has tumor promoting effects on a subset of DLBCL cell lines consistent with studies in mantle cell lymphoma where cell lines lacking expression of the endocannabinoid receptors were resistant to cell death induced by cannabinoids [26].

Serum concentrations in the culture media has profound effects on the results of cannabinoid-mediated cell growth *in vitro* [12, 31–34]. Consistent with previous studies [32], we found minimal effects of 2-AG with cells cultured in RPMI media supplemented with only 1% of CFBS; higher concentrations of CFBS (2.5% to 5%) are required for long-term growth of cells.

In summary, our study highlights the potential relevance of 2-AG in the pathogenesis and/or progression of DLBCL and its disparate influence on cell proliferation in various DLBCL cell lines. Further studies are needed to discern the possible role of 2-AG in the pathogenesis of obesity-related DLBCL.

## MATERIALS AND METHODS

### Study population

Serum was obtained from a subset of 100 (50 women, 50 men) HIV-negative, non-Hispanic white DLBCL cases and 100 controls frequency-matched for sex, age in 5-year groups, and BMI grouped per World Health Organization (WHO) categories, who participated in a population-based case-control study of NHL conducted in the San Francisco Bay Area [35]. DLBCL cases were grouped as either early stage (localized disease) or later stage (regional or remote disease). BMI was categorized using cut-points derived from WHO (http://apps.who.int/bmi/index.jsp?introPage=intro_3.html): underweight and healthy weight range, BMI > 18.5 - < 25 kg/m^2^; overweight, BMI ≥ 25 - < 30 kg/m^2^; and obese, BMI ≥ 30 kg/m^2^.

### Serum metabolite profiling of DLBCL cases and controls

Nonpolar lipid metabolites were extracted from 20 μL of serum in 3 ml of 2:1 chloroform:methanol and 1 ml of PBS with inclusion of internal standards dodecylglycerol (10 nmol) and pentadecanoic acid (10 nmol). Organic and aqueous layers were separated by centrifugation at 1000 x g for 5 min and the organic layer was collected, dried under a nitrogen stream and dissolved in 120 μl chloroform. Metabolites were then separated by reversed-phase liquid chromatography as previously described [36]. MS analysis was performed with an electrospray ionization (ESI) source on an Agilent 6430 QQQ LC-MS/MS. The capillary voltage was set to 3.0 kV, and the fragmentor voltage was set to 100 V. The drying gas temperature was 350°C, the drying gas flow rate was 10 l/min, and the nebulizer pressure was 35 psi. Untargeted LC-MS analysis was performed by scanning a mass range of *m/z* 50–1200 and data was exported as mz data files and uploaded to XCMS Online [37] (http://xcmsserver.nutr.berkeley.edu) to identify metabolites that changed between groups. Mass spectral peak adducts and isotopes were manually removed, while significantly and reproducibly changing masses were putatively identified by using the METLIN online database [38]. Metabolite identification was confirmed through single-reaction monitoring (SRM)- based targeted analyses based on retention time and ion fragmentation data of the transition from precursor to product ions at associated optimized collision energies acquired from synthetic standards as previously described [36]. Metabolites were quantified by integrating the area under the peak and normalized to internal standard values, and levels were expressed relative to controls.

### Reagents and chemical compounds

The following were purchased from Cayman (Ann Arbor, MI): 2-AG; the endocannabinoid receptor inhibitors, SR141716A (Rimonabant, CB1 inverse agonist), AM251 (CB1 antagonist) and SR144528 (CB2 inverse agonist); the endocannabinoid receptor activators, CP47497 (CB1 agonist), AM1241 (CB2 agonist), Win- 55–212-2 (CB1 and CB2 agonist) and CP55940 (CB1 and CB2 agonist); the selective and nonselective inhibitors for cyclooxygenase-1 and -2 (COX-1, COX-2), SC-560 (selective for COX-1), CAY10404 (selective for COX- 2), Ibuprofen (nonselective for COX-1 and COX-2); and the selective inhibitors for hydrolases of 2-AG, JZL 184 (selective for enzyme monoacylglycerol lipase, MAGL. Anti-bromodeoxyurine (BrdU) antibody was purchased from Roche Applied Science (Indianapolis, IN). The CellTiter 96 Non-Radioactive Cell proliferation Assay Kit™ was purchased from Promega (Madison, WI). Charcoal/dextran-treated fetal bovine serum (CFBS) was purchased from Hyclone (Logan, UT). DMEM, RPMI-1640, antibiotics (penicillin and streptomycin), and fetal bovine serum (FBS) were purchased from Atlanta Biologicals (Lawrenceville, GA).

### Cells and culture conditions

Sixteen germinal center and activated B-cell like DLBCL cell lines (Supplemental Table 1) were obtained from the following cell banks and no further authentication was required. Farage, Pfeiffer, SUDHL-4, SUDHL-6, and RL-2261 cell lines were obtained from American Type Culture Collection (ATCC, Masassas, VA). WSU-NHL and WSU-FSCCL DLBCL cell lines were obtained from Leibniz Institute DSMZ-German Collection of Microorganisms and Cell Culture (Braunschweig, Germany). The DLBCL cell line, Sci-1, was obtained from Sigma (St. Louis, MO). The DLBCL cell lines, OCI-LY-1, OCI-LY-2, OCI-LY-3, OCI-LY-10, OCI-LY-19, SUDHL-10 and RCK8, were obtained from Dr. L. Mernal- Mizrachi (Emory University, Atlanta, GA). The B-cell lymphoblastoid cell line, LBCL11832, was obtained from Coriell Cell Repositories (Coriell Institute, Camden, NJ). All DLBCL cell lines were cultured in RPMI-1640 supplemented with 10% FBS for less than 6 month after resuscitation. The cells were maintained under standard cell culture conditions at 37°C in 5% CO_2_ in a humid environment (Sanyo Electric Co., Japan). To minimize the influence of lipids, hormones and cytokines present in FBS, cells were cultured in RPMI media supplemented with CFBS for 24 hrs pre- and post-treatment.

### Lipidomic measurements of untreated lymphoblastoid cells and DLBCL cells

Lipid extraction and analysis of the metabolomes in untreated DLBCL cell lines and the lymphoblastoid cell line, LBCL11832, were conducted as previously described [36]. Briefly, lipid metabolites were extracted from cell pellets and subsequently analyzed using an SRM-based targeted approach. Lipid metabolite levels were measured in the Pfeiffer, Farage and LBCL11832 cell lines in RPMI media supplemented with 10% CFBS for 24 hours to minimize lipid metabolites in normal FBS in quintuple repeats.

### Cell proliferation assay

All 16 cell lines (15,000–20,000 cells/well) were treated with 2-AG at concentrations ranging from 1 nM to 10 μM for 4 days. After treatments, the number of live cells was measured at wavelength 570 nm using the MTT assay of mitochondrial enzymatic activity (CellTiter 96 Non-Radioactive Cell Proliferation Assay, Promega Corp., Madison, WI). In time-dependent studies, SUDHL-10, Pfeiffer, Sci-1, WSU-FSCCL and Farage cells were treated with 2-AG on days 1 through 4. For studies with agonists or antagonists of endocannabinoid receptors, the optimal maximum concentration of each compound was determined by comparing cell viability following treatments (1 nM to 10 μM) with that of the vehicle-treated control. SUDHL-10, Pfeiffer, Sci-1 and WSU-FSCCL cells were pretreated with each of the compounds at the optimal doses for 60 min, then 2-AG was added to a final concentration of 5 μM. For studies with selective inhibitors of metabolism for 2-AG, the optimal maximum concentration of each compound was determined by comparing cell viability following treatments (1 nM to 10 μM) with that of the vehicle-treated control. The four cell lines were pre-treated with the MAGL selective inhibitor, JZL184 (1 μM), the COX-1 selective inhibitor, SC-560 (1 μM); and the COX-1 and -2 nonselective inhibitor, Ibuprofen (40 μM), for 6 hours and then co-treated with 2-AG for 3 days. Acetonitrile, DMSO and ethanol were used as vehicles at concentrations ranging from 0.004% to 0.025% (v/v). Viability of treated cells was expressed as percent of vehicle-treated control. The fold change was the number of treated cells at each time point versus the initial number of cells at the start of treatment, reporting the mean of four repeats.

### Cell viability and cell counting

To verify the observed effects of 2-AG on proliferation via the MTT assay, after 2-AG treatment, cells were cultured for 3 days and collected from 96- well plates (quadruple repeats), stained with trypan blue and counted with an Auto T4 cellometer from Nexcelom Bioscience (Lawrence, MA). In all cases, experimental data were plotted as the percent change in treated cells as compared to vehicle-treated controls.

### Cell proliferation with 5-bromo-2′-deoxyuridine (BrdU) labeling

We evaluated the effect of 2-AG on cell cycle progression by BrdU incorporation in newly synthesized cellular DNA according to the manufacturer's recommendations using the Cell Proliferation ELISA, BrdU (colorimetric) Kit from Roche (Indianapolis, IN). Cells were cultured for 3 days with the indicated concentrations of 2-AG and labeled with 10 μM BrdU for 2 hours. The incorporation of BrdU was measured at 370 nm (490 nm as reference) using a microplate spectrophotometer, Synergy-H1 (BioTek, Winooski, VT). The percent change was calculated against the vehicle treatment and data were the average ± S.E. of four repeats in at least three independent experiments.

### Gene expression of endocannabinoid system in DLBCL cell lines

We used publicly available gene expression data on a subset of the DLBCL cell lines to determine if cannabinoid receptor-1 (*CNR1*), *CNR2*, G-protein-coupled receptor 55 (*GPR55*), fatty acid amide hydrolase (*FAAH*), monoacylglycerol lipase (*MAGL*), cyclooxygenase -1 (*COX1*) and cyclooxygenase -2 (*COX2*) gene expression correlated with the response of DLBCL cell lines to 2-AG. Data was downloaded from the Gene Expression Omnibus (GEO) repository (http://www.ncbi.nlm.nih.gov/geo) under accession number GSE15329.

### Statistical analysis

XCMS built-in statistical analysis incorporating R [37] was used to identify significantly changing ions at a particular retention time and *m/z* from all other ions in the sera. Two-tailed *t*-tests were used to identify significant changes between sample groups in normalized integrated values of individual ions at a given retention time detected by untargeted approaches in serum samples, and for normalized integrated values of metabolites detected by SRM-based targeted analyses in cell extracts. Experimental data from cell viability and proliferation assays were analyzed by the unpaired *t*-test and fitted with an exponential growth equation of Prism 6 for Windows from GraphPad Software (San Diego CA). Data are shown as means ±S.E. of values obtained from triplicate or quadruplicate experiments as indicated in each figure legend. In all cases, data were expressed as the percentage or fold increase of treated cells relative to vehicle-treated controls. A cut-off value of *p* < 0.05 was used to indicate statistical significance.

## SUPPLEMENTARY FIGURES AND TABLE



## Abbreviations

2-AG: 2-arachidonoylglycerol
2-OG: 2-oleoylglycerol
DLBCL: diffuse large B-cell lymphoma
DMSO: dimethylsulfoxide
ECS: endocannabinoid system
FAAH: fatty acid amide hydrolase
FBS: fetal bovine serum
FFA: free fatty acids
MAGL: monoacylglycerol lipase
PBS: phosphate buffered saline
RPMI: Roswell Park Memorial Institute Medium
