# Supplementary material for: The potential relevance of the endocannabinoid, 2-arachidonoylglycerol, in diffuse large B-cell lymphoma
Source: Oncoscience. 2016 Jan 30;3(1):31–41. doi: 10.18632/oncoscience.289 (PMC4751914; doi:10.18632/oncoscience.289)
Supplement: Supplementary file 1 [file oncoscience-03-031-s001.pdf]

SUPPLEMENTARY FIGURES AND TABLE

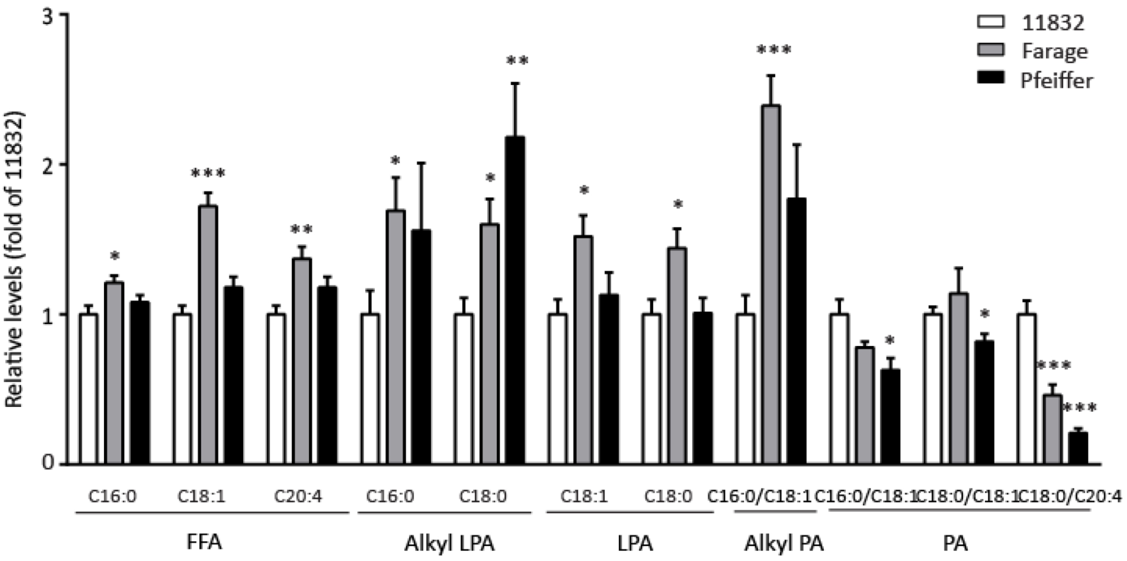

Supplementary Figure S1: Lipid levels of free fatty acids (FFA), alkyl lysophosphatidic acid (alkyl LPA), lysophosphatidic acid (LPA), alkyl phosphatidic acid (alkyl PA) and phosphatidic acid (PA) in cell lysates. Significantly higher levels of FFA in Farage cells including a 1.7-fold and 1.4-fold (P<0.01) increase in oleic acid and arachidonic acid, respectively. Levels of LPA species were elevated by 1.4 to 1.5-fold (P<0.05), and levels of alkyl-LPA species were elevated by 1.6 to 1.7 fold (P<0.05); C18:0 alkyl-LPA levels were increased by 2.2-fold in Pfeiffer cells (P<0.05). C16:0/C18:1 alkyl-PA levels were 2.4-fold higher in Farage cells (P<0.001). PA species were 2.2-fold lower in Farage (P<0.01) and 1.2 to 4.8-fold lower in Pfeiffer cells (P<0.05) compared to LBCL11832. Data from experiment performed in quadruplicates and expressed as fold change compared to LBCL11832. * P<0.05, ** P<0.01 and *** P<0.001

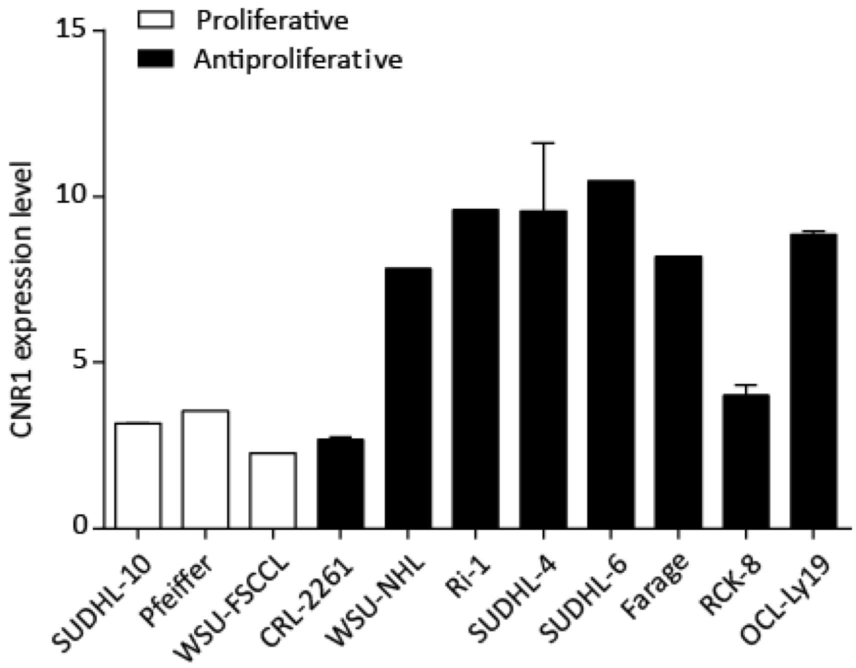

Supplementary Figure S2: In silico analysis of relative gene expression level of endocannabinoid receptor 1 (*CNR1*). Cell lines (white bars) are those proliferative upon 2-AG treatment (5-10 μM) and cell lines (black bars) are those anti-proliferative upon 2-AG treatment (5-10 μM)

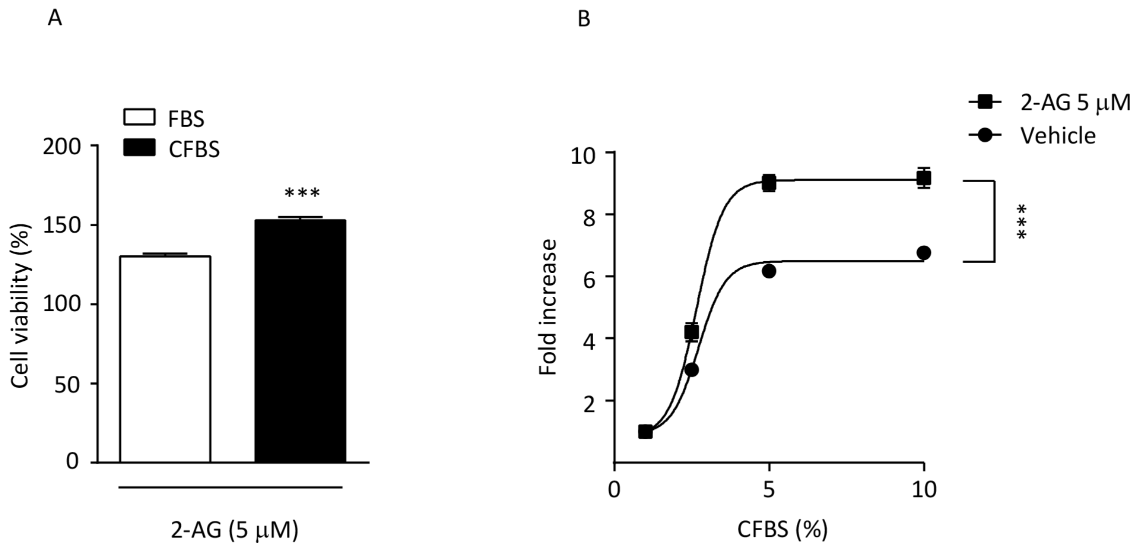

Supplementary Figure S3: Serum effects on the proliferation of DLBCL cell line SUDHL-10 treated with 2-AG. A. DLBCL cell line treated with 2-AG and cultured 4 days in the RPMI medium in the presence of 10% of FBS or CFBS. Data is presented as the percentage of the respective controls without 2-AG. **B.** DLBCL cell line treated with 2-AG and cultured for 4 days in the RPMI medium with 1, 2.5, 5, and 10% of FBS. Data are expressed as mean ± SE. * P<0.05, ** P<0.01, and *** P<0.001

Supplementary Table S1: DLBCL cell lines treated with 2-AG

Cell lines	Subtype
Pfeiffer	GCB
Sci-1	GCB
SUDHL-10	GCB
WSU-FSCCL	GCB
Farage	GCB
SUDHL-6	GCB
OCI-Ly3	ABC
OCI-Ly10	ABC
OCI-Ly1	GCB
CRL-2261	GCB
WSU-NHL	GCB
OCI-Ly2	GCB
OCI-Ly19	GCB
RCK8	GCB
Ri-1	ABC
SUDHL-4	GCB

GCB, germinal center B cell-like lymphoma; ABC, activated B cell-like lymphoma
